# Potential pregnancy days lost: an innovative measure of gestational age

**DOI:** 10.11606/s1518-8787.2020054002098

**Published:** 2020-08-24

**Authors:** Carmen Simone G Diniz, Jessica Reis-Queiroz, Carlos A. Kawai, Marcel Reis Queiroz, Eliana de Aquino Bonilha, Denise Yoshie Niy, Sonia Lansk, Brena Sena

**Affiliations:** I Universidade de São Paulo Faculdade de Saúde Pública Departamento de Saúde São PauloSP Brasil Universidade de São Paulo. Faculdade de Saúde Pública. Departamento de Saúde, Ciclos de vida e Sociedade. São Paulo, SP, Brasil; II São PauloSP Brasil Pesquisa Dias Potenciais de Gravidez Perdido. São Paulo, SP, Brasil; III São PauloSP Brasil Performance de Adquirencia e Antifraude Ecommerce. São Paulo, SP, Brasil; IV Universidade Nove de Julho Diretoria de saúde 3 São PauloSP Brasil Universidade Nove de Julho. Curso de Medicina. Diretoria de saúde 3. São Paulo, SP, Brasil; V Secretaria Municipal de Saúde de São Paulo São PauloSP Brasil Secretaria Municipal de Saúde de São Paulo. São Paulo, SP. Brasil.; VI ApiceON Project São PauloSP Brasil ApiceON Project, São Paulo, SP, Brasil; VII Departamento de Saúde prefeitura de Belo Horizonte Belo HorizonteMG Brasil Departamento de Saúde da prefeitura de Belo Horizonte. Belo Horizonte, MG, Brasil; VIII Harvard T.H. Chan School of Public Health BostonMA USA Harvard T.H. Chan School of Public Health. Boston, MA, USA

**Keywords:** Gestational Age, Infant, Newborn, growth & development, Labor, Induced, adverse effects, Cesarean Section, Iatrogenic Disease, Vital Statistics

## Abstract

In Brazil, the excess of interventions that anticipate childbirth, such as cesarean sections and labor inductions, has resulted in the shortening of pregnancy, with negative consequences on maternal-infant outcomes. This commentary presents a novel way to measure gestational age: the continuous variable “Potential pregnancy days lost.” Using data from the Live Birth Information System (SINASC), we counted the missing days between the period until childbirth and the average duration of pregnancy (280 days), or the lost weeks. This measure can be used as an outcome variable (socioeconomic-demographic characteristics of the mother, type of childbirth, financing, etc.) or exposure variable (for neonatal, infant, and maternal outcomes). The indicator can be used in municipal and national cohorts and intervention studies to analyze hospitals and regions. We discuss the limits and scope of gestational age measures and, given their inaccuracies, the importance of studying their trends.

## The Excess of Childbirth Interventions Has Reduced the Duration of Pregnancy

The health promotion of mothers and newborns and the health of next generations is a priority worldwide. One of the main concerns lies in the prevention of preterm birth, since these children usually present more health problems and disabilities, mostly affecting family well-being, in the health system and social costs. The more premature the children, the worse the health outcomes^1^. Depending on socioeconomic characteristics, health care and the quality of available data, prematurity rates range from less than 5% to more than 15% worldwide^2^.

For several decades, the “term” period (between 37 and 42 weeks of gestation) was treated as a homogeneous category, as it was believed that childbirth could occur or be initiated safely and without additional risks from its beginning (at 259 days, or 37 full weeks). Currently, we recognize that the traditional binary concept (term *versus* preterm) can mask the continuous effect of fetal immaturity as a predictor of negative outcome^3^. Thus, we are developing an innovative measure of gestational age (GA), a continuous variable named “potential pregnancy days lost” (PPDL), counting the missing days from the date of childbirth to the date when the average duration of pregnancy (280 days) would be completed, using data from birth records of the Live Birth Information System (SINASC).

Induction of labor and cesarean section are essential resources to achieve optimal maternal and neonatal morbidity and mortality rates, and they should be available in a timely manner for all women who need them. However, when the proportion of cesarean sections in a population exceeds the level between 10% and 15%, these intervention disadvantages tend to outweigh the potential benefits for mothers and infants, especially when performed before the spontaneous onset of labor and the 39 full weeks of gestation^4,5^. This is the case in Brazil, where GA in childbirth has decreased, as a result of the overestimation of the safety of interventions associated with the abbreviation of pregnancy (cesarean section and induction of labor)^6,7^.

The reasons why cesarean section is highly accepted in Brazil constitute a complex issue, including cultural, political, and economic aspects related to the barriers faced by the health system in offering a positive experience of childbirth, with humanized and evidence-based care. The high financial convenience of elective cesarean section, both for health professionals and institutions^6,7^, also has its influence. In Brazil, childbirth before physiological maturity can occur due to clinical and socioeconomic factors, iatrogenic causes or a combination of factors.

## Shortening of pregnancy as a Public Health Issue and the New Classification of GA

**Figure 1:**
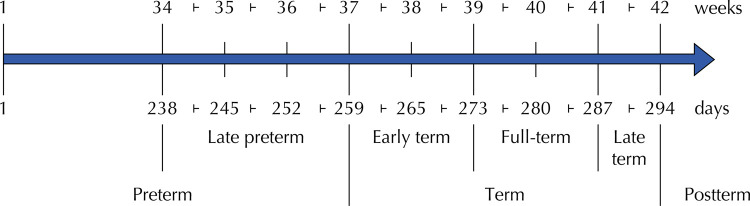
New gestational age classification, according to the American College of Obstetricians and Gynecologists4.

In the last two decades, robust evidence has indicated that being born at “early term” (37 0/7 weeks to 38 6/7 weeks) is associated with health outcomes more similar to those born at “late-preterm” (34 0/7 to 36 6/7) than with those born with more than 39 weeks^4,8^. Based on this innovative interpretation, a new categorical classification was adopted, subdividing the period of “term” into “early term”, “full term” (39 0/7 to 40 6/7), and “late term” (41 0/7 to 41 6/7),^4^ according to Figure 1.

An analysis of data from the Birth in Brazil National Inquiry (2011–2012), with 23,940 mother-baby pairs, shows that early term childbirth was associated with an increased risk of neonatal death, neonatal ICU admission, oxygen therapy, hypoglycemia, transient tachypnea, need for phototherapy and lower chances of being breastfed in the first hour of life or during hospitalization^9^. Even with the confirmed pulmonary maturity, early term childbirth is associated with worse neonatal respiratory and hepatic outcomes compared with full-term births^10,11^. This increase in preventable morbidity and mortality burdens financially and logistically a health system already quite deficient.

Regarding long-term consequences of perinatal interventions, solid pieces of evidence show that cesarean section is associated with an increased risk of chronic diseases in childhood, such as asthma, diabetes, obesity, hypertension, arthritis, neurological problems, allergies, and certain types of cancer such as leukemia^11^. Excessive use of cesarean section and induction of labor also increases the risks of maternal morbidity and mortality, directly affecting mothers and their children, indirectly^11,22^.

There is growing evidence about an inverted dose-response effect between GA in full-term newborns and the risk of developmental delays^23^. Studies show that, even controlling confounding variables related to sociodemographic aspects and maternal morbidity, an infant born by elective cesarean section, even in the term period, is associated with worse school performance in language and mathematics^24^. A systematic review of 61 studies covering more than 20 million childbirths showed that caesarean section delivery was significantly associated with autism spectrum disorder and attention-deficit hyperactivity, regardless of cesarean section modality, compared with vaginal birth^23^.

Unnecessary shortening of pregnancy affects neonatal mortality and neonatal and maternal morbidity, as well as long-term effects on chronic diseases in children, including neurocognitive development. Thus, this is a relevant and underestimated public health issue, considering its high prevalence, severity, and avoidability (possibility of control) in the Brazilian case.

A total of three interrelated mechanisms are involved in health complications resulting from the excessive use of interventions related to pregnancy abbreviation , even in full-term births (37 to 42 weeks)^9,10^. Firstly, considering that biological signs of fetal maturity trigger spontaneous labor, elective cesarean section without labor, as well as induction of labor, prevent the baby from achieving this maturity to born, and such techniques should be used with caution, when the benefits clearly outweigh risks^3,9^.

Secondly, the hours of spontaneous labor promote epigenetic expression of genes associated with fetal transition to extrauterine life^10^. Nowadays, it is recognized that complex neuroendocrine processes, with the release of catecholamines, oxytocin, and endorphins during the different stages of labor, prepare the immune, respiratory, and gastrointestinal systems of the fetus, in addition to protecting the child’s brain from birth stress, reducing vulnerability to hypoxia and fetal distress, resulting in a safer fetal-neonatal transition^3,11,13^. The physiological release of oxytocin and endorphins is also related to a more comfortable childbirth for the mother and baby, reducing the risk of maternal hemorrhage and facilitating the initiation of breastfeeding^10^.

Thirdly, in a cesarean section, for the children do not pass through the birth canal, they are deprived of contact with the mother’s vaginal bacteria, which is currently recognized as a key element for a healthy colonization of the child’s microbiome^10^. This early contact is associated with a healthier activation of the immune system, better response to infections, and less inflammatory metabolism, leading to greater resilience to chronic diseases in childhood and adulthood^11^. In an elective cesarean section, these three protective mechanisms will be impaired.

## Advantages and Limitations of GA Measurement in Days

The mean GA of the population at birth, based on spontaneous childbirth, is considered internationally as 40 weeks (280 days) from the first day of the last menstrual period (LMP)^25^. The estimation of GA can also be carried out by ultrasound, whose accuracy is good at the beginning of pregnancy (considered as the “gold standard” measure), but it can reach three weeks of error or more in the final months. After childbirth, GA can also be estimated by physical examination of the newborn^19,25^. As a reflection of the international change in the understanding of the risks associated with the anticipation of childbirth even within the term period, since 2011 SINASC allows to estimate the GA in weeks and days, based on the date of the last menstrual period, or other estimation methods. Thus, GA can be used as a continuous variable, if there is good completeness, or corrected by linkage of databases or imputation.

The estimate of GA at birth is innacurate, for the reasons mentioned and for the individual variability of fetal maturation. However, in addition to the accuracy of GA, considering public health, it is relevant to study its temporal changes and trends in the population and associated factors.

Although it is possible to estimate GA in days, in practice we observe a tendency to recategorize GA in weeks of gestation and in the term interval previously defined (37 to 42 weeks), limiting the potential to increase the granularity of data^6^. A qualitative component of this research is exploring the problems related to filling out the form, by ethnographic observation and interviews, to better understand the systematic biases in the information of GA in the declarations of live births and the logic behind them.

It is also advantageous to increase the granularity of data using GA weeks or term fractions (early, full, and late), especially when data in days, based on LMP, are not available. Data from the Birth in Brazil survey, as well as from the São Paulo municipal SINASC, show the apex of the GA curve for vaginal deliveries corresponds to 39 weeks, whereas for cesarean section it is 38 weeks,^6,7^ trends that also apply at national level^27^. This is an “inversion of the expected disparity,” since socioeconomic factors are decisive for the best maternal-infant outcomes, and historically it is expected that those born from more educated mothers and private sector users are born with higher GA^28^.

## An Innovative Indicator, Based on a New Interpretation of Available Data, to Subsidize Public Policies

From the moment we recognize that each day of pregnancy counts on perinatal health and well-being, we should move towards a “harmfree care”^29^, making visible not only the number of days and weeks of gestation lost, but also how this loss is potentially harmful. As data often present collection errors, information can be adjusted by considering the parameters of normal distributions, presented by subgroups with GA measured by LMP and “other” methods, as it was successfully performed using data from Birth in Brazil^30^.

This research aims to develop and test the indicator “potential pregnancy days lost.” We are starting with the SINASC database of the Municipal Health Department of São Paulo, in partnership with the management of this system. We have created new databases using LMP information, transforming GA into days, and potential pregnancy days lost. These databases are linked to the Brazilian Mortality Information System (MIS), enabling the study of the relationships between GA in days with infant and maternal death.

Considering that part of the database does not have LMP data, besides experimenting the imputation of the missing GA data in days, we have used the available information of pregnancy dating by other methods (ultrasound and physical examination), increasing the granularity of GA using the measurement in weeks and fractions of the term (early, full, late). We are developing machine learning resources to create predictive models related to maternal and neonatal risks, using PPDL as a continuous variable, also testing gestational age values with different granularities.

This project is being conducted in partnership with public managers to provide an alignment of their outcomes to the needs of municipal management and services. Finally, it is expected to introduce and make current the PPDL indicator in the practice and training of health professionals, supporting a safer, more respectful perinatal care, based on the best care practices.

## References

[1] 1. Raju TNK, Buist AS, Blaisdell CJ, Moxey-Mims M, Saigal S. Adults born preterm: a review of general health and system-specific outcomes. Acta Paediatr. 2017;106(9):1409-37. 10.1111/apa.13880 28419544

[2] 2. Chawanpaiboon S, Vogel JP, Moller AB, Lumbiganon P, Petzold M, Hogan D, et al. Global, regional, and national estimates of levels of preterm birth in 2014: a systematic review and modelling analysis. Lancet Glob Health. 2019;7(1):e37-46. 10.1016/S2214-109X(18)30451-0 PMC6293055 30389451

[3] 3. Hillman NH, Kallapur SG, Jobe AH. Physiology of transition from intrauterine to extrauterine life. Clin Perinatol. 2012;39(4):769-83. 10.1016/j.jped.2016.08.004 PMC3504352 23164177

[4] 4. American College of Obstetricians and Gynecologists Committee on Obstetric Practice Society for Maternal-Fetal Medicine. Definition of Term Pregnancy. Washington, DC: ACOG; 2013. (Committee Opinion, 579)

[5] 5. Organização Mundial de Saúde. Programa de Reprodução Humana. Declaração da OMS sobre Taxas de Cesáreas. Genebra: OMS; 2015 [cited 2015 Feb 4]. Available from: https://apps.who.int/iris/bitstream/handle/10665/161442/WHO_RHR_15.02_por.pdf;jsessionid=9A1A1E13AAB031B26242C70B1DB13BB8?sequence=3

[6] 6. Diniz CSG, Miranda MJ, Reis-Queiroz J, Queiroz MR, Salgado HO. Why do women in the private sector have shorter pregnancies in Brazil? Left shift of gestational age, caesarean section and inversion of the expected disparity. J Hum Growth Dev. 2016 26(1):33-40. 10.7322/jhgd.113712

[7] 7. Raspantini PR, Miranda MJ, Silva ZP, Alencar GP, Diniz SG, Almeida MF de. O impacto do tipo de hospital e tipo de parto sobre a idade gestacional ao nascer no Município de São Paulo, 2013-2014. Rev Bras Epidemiol. 2016;19(4):878-82. 10.1590/1980-5497201600040016 28146175

[8] 8. World Health Organization; March of Dimes; The Partnership for Maternal, Newborn & Child Health; Save the Children. Born too soon: the global action report on preterm birth. Geneva: WHO; 2012 [cited 2015 Feb 4]. Available from: https://www.who.int/maternal_child_adolescent/documents/born_too_soon/en/

[9] 9. Dahlen HG, Kennedy HP, Anderson CM, Bell AF, Clark A, Foureur M, et al. The EPIIC hypothesis: intrapartum effects on the neonatal epigenome and consequent health outcomes. Med Hypotheses. 2013;80(5):656-62. 10.1016/j.mehy.2013.01.017 PMC3612361 23414680

[10] 10. Buckley SJ. Executive summary of hormonal physiology of childbearing : evidence and implications for women, babies, and maternity care. J Perinat Educ. 2015;24(3):145-53. 10.1891/1058-1243.24.3.145. PMC4720867 26834435

[11] 11. Sandall J, Tribe RM, Avery L, Mola G, Visser GH, Homer CS, et al. Short-term and long-term effects of caesarean section on the health of women and children. Lancet. 2018;392(10155):1349-57. https://doi.org/10.1016/S0140-6736(18)31930-5 10.1016/S0140-6736(18)31930-5 30322585

[12] 12. Martínez-Nadal S, Demestre X, Raspall F, Álvarez JA, Elizari MJ, Vila C, et al. Morbilidad neonatal en los recién nacidos a término precoz. An Pediatr. 2014;81(1):39-44. 10.1016/j.anpedi.2013.10.015 24286869

[13] 13. Dahlen HG, Downe S, Kennedy HP, Foureur M. Is society being reshaped on a microbiological and epigenetic level by the way women give birth? Midwifery. 2014;30(12):1149-51. 10.1016/j.midw.2014.07.007 25128348

[14] 14. Gutvirtz G, Wainstock T, Sheiner E, Landau D, Walfisch A. Pediatric cardiovascular morbidity of the early term newborn. J Pediatr. 2018;194:81-6. 10.1016/j.jpeds.2017.09.060 29129352

[15] 15. Cascaes AM, Gauche H, Baramarchi FM, Borges CM, Peres KG. Prematuridade e fatores associados no Estado de Santa Catarina, Brasil, no ano de 2005: análise dos dados do Sistema de Informações sobre Nascidos Vivos. Cad Saude Publica. 2008;24(5):1024-32. 10.1590/S0102-311X2008000500009 18461231

[16] 16. Kilsztajn S, Rossbach A, Carmo MSN, Sugahara GTL. Assistência pré-natal, baixo peso e prematuridade no Estado de São Paulo, 2000. Rev Saude Publica. 2003;37(3):303-10. 10.1590/S0034-89102003000300007 12792680

[17] 17. Sclowitz IKT, Santos IS. Fatores de risco na recorrência do baixo peso ao nascer, restrição de crescimento intra-uterino e nascimento pré-termo em sucessivas gestações: um estudo de revisão. Cad Saude Publica. 2006;22(6):1129-36. 10.1590/S0102-311X2006000600002 16751952

[18] 18. Leal MC, Pereira APE, Domingues RMSM, Theme Filha MM, Dias MAB, Nakamura-Pereira M, et al. Intervenções obstétricas durante o trabalho de parto e parto em mulheres brasileiras de risco habitual. Cad Saude Publica. 2014;30 Supl 1:S17-32. 10.1590/0102-311X00151513

[19] 19. Leung JYY, Lam HS, Leung GM, Schooling CM. Gestational age, birthweight for gestational age, and childhood hospitalisations for asthma and other wheezing disorders. Paediatr Perinat Epidemiol. 2016;30(2):149-59. 10.1111/ppe.12273 26739588

[20] 20. Thomopoulos TP, Skalkidou A, Dessypris N, Chrousos G, Karalexi MA, Karavasilis TG, et al. Prelabor cesarean delivery and early-onset acute childhood leukemia risk. Eur J Cancer Prev. 2016;25(2):155-61. 10.1097/CEJ.0000000000000151 25793919

[21] 21. Paz Levy D, Sheiner E, Wainstock T, Sergienko R, Landau D, Walfisch A. Evidence that children born at early term (37-38 6/7 weeks) are at increased risk for diabetes and obesity-related disorders. Am J Obstet Gynecol. 2017;217(5):588.e1-588.e11. 10.1016/j.ajog.2017.07.015 28729012

[22] 22. Leal MC, Esteves-Pereira AP, Nakamura-Pereira M, Domingues RMSM, Dias MAB, Moreira ME, et al. Burden of early-term birth on adverse infant outcomes: a population-based cohort study in Brazil. BMJ Open. 2017;7(12):e017789. 10.1136/bmjopen-2017-017789 PMC5770827 29284716

[23] 23. Zhang T, Sidorchuk A, Sevilla-Cermeño L, Vilaplana-Pérez A, Chang Z, Larsson H, et al. Association of cesarean delivery with risk of neurodevelopmental and psychiatric disorders in the offspring: a systematic review and meta-analisys. JAMA Netw Open. 2019;2(8):e1910236. 10.1001/jamanetworkopen.2019.10236 PMC6716295 31461150

[24] 24. Polidano C, Zhu A, Bornstein JC. The relation between cesarean birth and child cognitive development. Sci Rep. 2017;7:11483. 10.1038/s41598-017-10831-y PMC5597642 28904336

[25] 25. Bergsjo P, Denman DW 3rd, Hoffman HJ, Meirik O. Duration of human singleton pregnancy. A population-based study. Acta Obstet Gynecol Scand. 1990;69(3):197-207. 10.3109/00016349009028681 2220340

[26] 26. Pereira APE, Leal MC, Gama SGN, Domingues RMSM, Schilithz AOC, Bastos MH. Determining gestational age based on information from the Birth in Brazil study. Cad Saude Publica. 2014;30 Supl 1:S59-70. 10.1590/0102-311X00160313 25167191

[27] 27. Barros FC, Rabello Neto DL, Villar J, Kennedy SH, Silveira MF, Diaz-Rossello JL, et al. Caesarean sections and the prevalence of preterm and early-term births in Brazil: secondary analyses of national birth registration. BMJ Open. 2018;8(8). 10.1136/bmjopen-2018-021538 PMC6078248 30082353

[28] 28. Diniz SG, D’Oliveira AFPL, Lansky S. Equity and women’s health services for contraception, abortion and childbirth in Brazil. Reprod Health Matters. 2012;20(40):94-101. 10.1016/S0968-8080(12)40657-7 23245414

[29] 29. United Kingdom National Health Services. NHS Safety Thermometer. London (UK): NHS; 2013 [cited 2015 Feb 4]. Available from: www.safetythermometer.nhs.uk

[30] 30. Queiroz JR. Dias de gravidez potencialmente perdidos: um novo olhar sobre a idade gestaciona [tese]l. São Paulo: Escola de Enfermagem da Universidade de São Paulo; 2018.

