# Supplementary material for: Potential pregnancy days lost: an innovative measure of gestational age
Source: Rev Saude Publica. 2020 Aug 24;54:88. doi: 10.11606/s1518-8787.2020054002098 (PMC7446763; doi:10.11606/s1518-8787.2020054002098)
Supplement: Translation

# Translation: Dias potenciais de gravidez perdidos: uma medida inovadora da idade gestacional

**Authors:** Carmen Simone G Diniz, Jessica Reis-Queiroz, Carlos A. Kawai, Marcel Reis Queiroz, Eliana de Aquino Bonilha, Denise Yoshie Niy, Sonia Lansk, Brena Sena

**Affiliations:** I Brasil Universidade de São Paulo. Faculdade de Saúde Pública. Departamento de Saúde, Ciclos de vida e Sociedade. São Paulo, SP, Brasil; II Brasil Pesquisa Dias Potenciais de Gravidez Perdido. São Paulo, SP, Brasil; III Brasil Performance de Adquirencia e Antifraude Ecommerce. São Paulo, SP, Brasil; IV Brasil Universidade Nove de Julho. Curso de Medicina. Diretoria de saúde 3. São Paulo, SP, Brasil; V Brasil Secretaria Municipal de Saúde de São Paulo. São Paulo, SP. Brasil.; VI Brasil ApiceON Project, São Paulo, SP, Brasil; VII Brasil Departamento de Saúde da prefeitura de Belo Horizonte. Belo Horizonte, MG, Brasil; VIII USA Harvard T.H. Chan School of Public Health. Boston, MA, USA

**Keywords:** Idade Gestacional, Recém-Nascido, crescimento & desenvolvimento, Trabalho de Parto Induzido, efeitos adversos, Cesárea, Doença Iatrogênica, Estatísticas Vitais

## Abstract

No Brasil, o excesso de intervenções que antecipam o parto, como cesarianas e induções, tem resultado no encurtamento da gestação, com consequências negativas nos desfechos materno-infantis. Neste comentário apresentamos uma medida inovadora da idade gestacional que estamos desenvolvendo: a variável contínua “dias potenciais de gravidez perdidos”. Utilizando dados do Sistema de Informações de Nascidos Vivos, contamos, quando possível, os dias faltantes entre o período até o nascimento e a duração média da gestação (280 dias), ou então as semanas perdidas. Essa medida pode ser usada como variável desfecho (de características socioeconômico-demográficos da mãe, do tipo de parto, de financiamento etc.) ou de exposição (para desfechos neonatais, infantis e maternos). O indicador pode ser usado em coortes municipais e nacionais e em estudos de intervenção, para analisar hospitais e territórios. Discutimos os limites e alcances das medidas de idade gestacional e, diante de suas imprecisões, a importância de estudar suas tendências.

## O Excesso de Intervenções no Parto Tem Reduzido a Duração da Gravidez

A promoção da saúde de mães e recém-nascidos, e assim a saúde das próximas gerações, é uma prioridade de saúde pública em todo o mundo. Uma das principais preocupações consiste em prevenir o nascimento pré-termo, uma vez que essas crianças tendem a apresentar mais problemas de saúde e deficiências, com grande impacto no bem-estar familiar, no sistema de saúde e em custos sociais. Quanto mais prematuras as crianças, piores os resultados de saúde^1^. Dependendo das características socioeconômicas, da assistência à saúde e da qualidade dos dados disponíveis, as taxas de prematuridade variam de menos de 5% a mais de 15% no plano internacional^2^.

Por várias décadas, o período de “termo” (compreendido entre 37 e 42 semanas de gestação) foi tratado como uma categoria homogênea, pois se acreditava que o nascimento poderia ocorrer ou ser provocado de maneira segura e sem riscos adicionais a partir do seu início (aos 259 dias, ou 37 semanas completas). Atualmente reconhecemos que o conceito binário tradicional (termo *versus* pré-termo) pode mascarar o efeito contínuo da imaturidade fetal como preditora de resultados negativos^3^. Por esse motivo, estamos desenvolvendo uma medida inovadora de idade gestacional (IG), uma variável contínua chamada “dias potenciais de gravidez perdidos” (DPGP), contando os dias faltantes desde a data do nascimento até a data em que se completaria a duração média da gestação (280 dias), usando dados de registros de nascimentos do Sistema de Informações de Nascidos Vivos (Sinasc).

A indução do trabalho de parto e a cesariana são recursos essenciais para atingir taxas ótimas de morbimortalidade materna e neonatal, e devem estar disponíveis oportunamente para todas as mulheres que delas precisem. Todavia, quando a proporção de cesarianas em uma população ultrapassa o patamar de 10% a 15%, suas desvantagens tendem a superar os potenciais benefícios para mães e bebês, especialmente quando realizadas antes do início espontâneo do trabalho de parto e das 39 semanas completas de gestação^4,5^. Esse é o caso do Brasil, onde a IG no parto tem diminuído, como resultado da superestimação da segurança de intervenções associadas com a abreviação da gravidez (cesariana e indução do trabalho de parto)^6,7^.

Os motivos pelos quais a cesariana tem uma aceitação tão alta no Brasil conformam um problema complexo, que inclui aspectos culturais, políticos e econômicos relacionados às dificuldades do sistema de saúde para oferecer uma experiência positiva de parto, com cuidado humanizado e baseado em evidências. Também pesam a elevada conveniência financeira da cesariana eletiva, tanto para profissionais de saúde como para as instituições^6,7^. No Brasil, o nascimento antes da maturidade fisiológica pode ocorrer por fatores clínicos e socioeconômicos, por causas iatrogênicas ou por uma combinação de fatores.

## O Encurtamento da Gravidez como Problema de Saúde Pública e a Nova Classificação da IG

Nas últimas duas décadas, evidências robustas emergiram indicando que nascer a “termo precoce” (37 0/7 semanas a 38 6/7 semanas) está associado com resultados de saúde mais parecidos com os dos nascidos de “pré-termo tardio” (34 0/7 a 36 6/7) do que com os nascidos com mais de 39 semanas^4,8^. Com base nessa interpretação inovadora, uma nova classificação categórica foi adotada, subdividindo o período de “termo” em “termo precoce”, “termo pleno” (39 0/7 a 40 6/7) e “termo tardio” (41 0/7 a 41 6/7)^4^, conforme a Figura 1.

**Figura 1:** Nova classificação de idade gestacional, de acordo com a *American College of Obstetricians* and Gynecologists4.

Uma análise dos dados do Inquérito Nacional Nascer no Brasil (2011–2012), com 23.940 pares mãe-bebê, mostra que o nascimento a termo precoce esteve associado com um aumento de risco de morte neonatal, admissão em UTI neonatal, oxigenioterapia, hipoglicemia, taquipneia transitória, necessidade de fototerapia e chances menores de ser amamentado na primeira hora de vida ou durante a internação^9^. Mesmo com a maturidade pulmonar confirmada, o parto a termo precoce é associado com piores resultados respiratórios e hepáticos neonatais em comparação com nascidos a termo pleno^10,11^. Esse aumento da morbimortalidade evitável onera financeira e logisticamente ainda mais um sistema de saúde já bastante deficitário.

Em termos de consequências em longo termo de intervenções perinatais, evidências sólidas mostram que a cesárea está associada a um risco aumentado de doenças crônicas na infância, como asma, diabetes, obesidade, hipertensão, artrite, problemas neurológicos, alergias e certos tipos de câncer como leucemia^11^. O uso excessivo de cesárea e indução também aumenta os riscos de morbidade e mortalidade materna, impactando as mães diretamente e seus filhos indiretamente^11,22^.

Há evidências crescentes de um efeito dose-resposta invertido entre IG em recém-nascidos a termo e o risco de atrasos no desenvolvimento^23^. Estudos mostram que, mesmo controlando variáveis de confusão relacionadas a aspectos sociodemográficos e à morbidade materna, ter nascido por uma cesariana eletiva, ainda que no período de termo, está associado com pior desempenho escolar em linguagem e matemática^24^. Uma recente revisão sistemática de 61 estudos envolvendo mais de 20 milhões de partos mostrou que nascer por cesariana foi significativamente associado ao distúrbio do espectro do autismo e ao déficit de atenção e hiperatividade, independentemente da modalidade de parto cesáreo, em comparação com o parto vaginal^23^.

O encurtamento desnecessário da gravidez tem efeito sobre a mortalidade neonatal e a morbidade neonatal e materna, além de efeitos de longo prazo sobre doenças crônicas nos nascidos, incluindo o desenvolvimento neuro-cognitivo. Pela sua alta prevalência no caso brasileiro, gravidade e evitabilidade (possibilidade de controle), trata-se de um relevante e subestimado problema de saúde pública.

Três mecanismos inter-relacionados estão envolvidos nas complicações de saúde decorrentes do uso excessivo de intervenções que resultam na abreviação da gravidez, mesmo nos nascidos a termo (37 a 42 semanas)^9,10^. Primeiro, considerando que sinalizações biológicas da maturidade fetal desencadeiam o trabalho de parto espontâneo, a cesariana eletiva fora de trabalho de parto, assim como a indução, impedem que o bebê atinja essa prontidão para nascer, e deveriam ser usadas com cautela, quando os benefícios claramente compensam os riscos^3,9^.

Segundo, as horas de trabalho de parto espontâneo promovem a expressão epigenética dos genes associados com a transição fetal para a vida extrauterina^10^. Atualmente se reconhece que complexos processos neuroendócrinos, com a liberação de catecolaminas, ocitocina e endorfinas durante as diferentes fases do trabalho de parto, preparam os sistemas imune, respiratório e gastrointestinal do feto, além de proteger o cérebro da criança do estresse do nascimento, reduzindo a vulnerabilidade à hipóxia e o sofrimento fetal, resultando em uma transição fetal-neonatal mais segura^3,11,13^. A liberação fisiológica de ocitocina e endorfinas também está relacionada com um parto mais confortável para a mãe e para o bebê, reduzindo o risco de hemorragia materna e facilitando o início da amamentação^10^.

Terceiro, em uma cesárea, ao não passar pelo canal de parto, a criança é privada do contato com as bactérias vaginais da mãe, que atualmente é reconhecido como elemento-chave para uma colonização saudável do microbioma da criança^10^. Esse contato precoce está associado com uma ativação mais saudável do sistema imune, uma resposta melhor a infecções e um metabolismo menos inflamatório, levando a uma maior resiliência a doenças crônicas na infância e vida adulta^11^. Em uma cesárea eletiva, esses três mecanismos de proteção serão prejudicados.

## Vantagens e Limitações da Medida de IG em Dias

A IG média da população ao nascer, baseada nos partos espontâneos, é considerada internacionalmente como 40 semanas (280 dias) a partir do primeiro dia da última menstruação (DUM)^25^. O cálculo da IG pode ser feito também por ultrassom, cuja precisão é boa no início da gestação (considerado como a medida “padrão-ouro”), mas pode atingir três semanas de erro ou mais nos meses finais. Depois do parto, a IG também pode ser estimada pelo exame físico do recém-nascido^19,25^. Como reflexo da mudança internacional na compreensão dos riscos associados com a antecipação do parto mesmo dentro do período de termo, desde 2011 o Sinasc permite calcular a IG em semanas e dias, com base na data da última menstruação, ou outros métodos de estimação. Isso permite seu uso como uma variável contínua, se há boa completitude, ou corrigida por *linkage* (relacionamento) de bases de dados ou imputação.

A estimativa da IG ao nascimento é imprecisa, pelas razões acima descritas, e também pela variabilidade individual do amadurecimento fetal. Porém, além da exatidão da IG, em termos de saúde pública importa o estudo das suas mudanças temporais e tendências na população e dos fatores associados.

Apesar de ser possível calcular a IG em dias, na prática observa-se uma tendência de recategorizar a IG em semanas de gestação e no intervalo de termo definido anteriormente (37 a 42 semanas), limitando o potencial de ampliar a granularidade dos dados^6^. Um componente qualitativo desta pesquisa está explorando os problemas do preenchimento da ficha, por observação etnográfica e entrevistas, para melhor entender os vieses sistemáticos na informação da IG nas declarações de nascidos vivos e a lógica por trás deles.

Também é vantajoso aumentar a granularidade dos dados usando semanas de IG ou as frações do termo (precoce, pleno e tardio), principalmente quando os dados em dias, com base na DUM, não estão disponíveis. Dados do inquérito Nascer no Brasil, assim como do Sinasc municipal de São Paulo, mostram que o ápice da curva da IG para partos vaginais corresponde a 39 semanas, enquanto para a cesárea é de 38 semanas^6,7^, tendências que também se aplicam no nível nacional^27^. Isso é uma “inversão da disparidade esperada”, uma vez que os fatores socioeconômicos são decisivos para os melhores desfechos materno-infantis, e historicamente se espera que os nascidos de mães mais escolarizadas e usuárias do setor privado nasçam com IG maior^28^.

## Um Indicador Inovador, Baseado em uma Nova Interpretação de Dados Disponíveis, para Subsidiar Políticas Públicas

A partir do momento em que se reconhece que cada dia de gestação conta na saúde e no bem-estar perinatais, deve-se avançar no sentido de um “cuidado livre de danos”^29^, tornando visível não apenas o número de dias e semanas de gestação perdidos, mas também o quanto essa perda é potencialmente danosa. Como os dados frequentemente apresentam erros de coleta, a informação pode ser ajustada ponderando-se os parâmetros das distribuições normais, apresentada por subgrupos com IG medida pela DUM e por “outros” métodos, como se realizou com sucesso usando dados do Nascer no Brasil^30^.

Esta pesquisa tem como objetivo desenvolver e testar o indicador “dias potenciais de gravidez perdidos”. Estamos iniciando com a base de dados do Sinasc da Secretaria Municipal de Saúde de São Paulo, em parceria com a gerência desse sistema. Criamos bases de dados novas usando informações da DUM, transformando a IG em dias e em dias potenciais de gravidez perdidos. Essas bases estão vinculadas com o Sistema de Informação sobre Mortalidade (SIM), permitindo o estudo das relações entre IG em dias com a morte infantil e materna.

Uma vez que parte da base não tem dados da DUM, além experimentar a imputação dos dados faltantes da IG em dias, temos usado a informação disponível de datação da gravidez por outros métodos (ultrassonografia e exame físico), aumentando a granularidade da IG usando a medida em semanas e em frações do termo (precoce, pleno, tardio). Estamos desenvolvendo recursos de *machine learning* para a criação de modelos preditivos relativos a riscos maternos e neonatais, usando os DPGP como variável contínua, testando também medidas de idade gestacional com diferentes granularidades.

Este projeto está sendo conduzido em parceria com gestores públicos, de forma a propiciar um alinhamento dos seus desfechos às necessidades da gestão municipais e dos serviços. Por fim, espera-se introduzir e tornar corrente o indicador de DPGP na prática e na formação de profissionais de saúde, ajudando a promover um cuidado perinatal mais seguro, respeitoso e baseado nas melhores práticas de cuidado.
